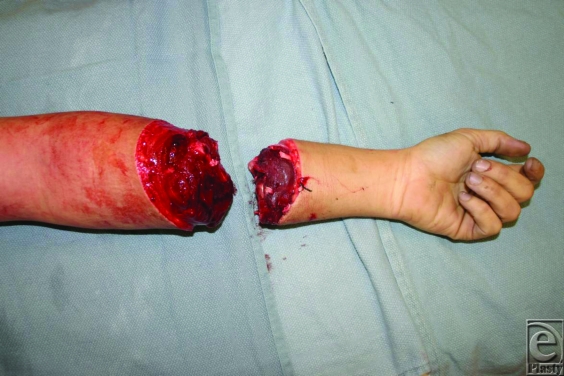# Major Limb Replantation

**Published:** 2011-01-11

**Authors:** Janet H. Yueh, Eran D. Bar-Meir, Eric C. Liao, Bernard T. Lee

**Affiliations:** ^a^Department of Surgery, Division of Plastic and Reconstructive Surgery, University of Dentistry and Medicine–New Jersey, Newark; ^b^Beth Israel Deaconess Medical Center, Boston, MA; ^c^Massachusetts General Hospital, Harvard Medical School, Boston

## DESCRIPTION

A 32-year-old right-hand-dominant man presented with a traumatic guillotine-type amputation of the left forearm at the junction of the musculotendinous transition. The amputated part was well preserved, with a minimal zone of injury. The patient sustained no other injuries and he was cleared for surgery by the trauma service. He was taken to the operating room for replantation of the amputated part.

## QUESTIONS

1. **What are the major concerns with replantation of a major extremity?**
2. **What metabolic changes are seen after reperfusion of a large amputated part?**
3. **What is the prognosis for patients with replantation at this level?**

## DISCUSSION

Since the first successful limb transplant in 1962, the literature continues to support replantation as the best method to restore function to an amputated extremity. Replantation of a limb continues to be a technically demanding procedure. While an amputation is the sum of a vascular injury, an open fracture, a soft tissue injury, and a nerve injury, reattachment of the individual parts can result in severe morbidity during and after surgery.

When undertaking upper extremity replantation, one must be mindful of immediate and future goals, including arm revascularization and future recovery of function. Whether or not the replantation should be attempted is clearly dependent on the circumstances of the trauma and patient. In cases where replantation is not possible, one should always attempt to salvage enough of the proximal extremity to fit with a prosthesis.

Only after the patient is stabilized by a multidisciplinary trauma team can limb salvage be considered. For efficiency in the operating room, multiple teams after often necessary for a major limb replantation. The major goal is to reestablish limb perfusion and minimize ischemia time. Replantation usually begins with bony fixation followed by arterial repair. Bone shortening is crucial to achieve tension-free repairs of other structures. In the presented case, a venous coupling device was used to perform 6 different anastomoses; this allowed for maximal venous outflow and can be performed rapidly. Postoperative venous congestion and swelling often require prophylactic fasciotomies to prevent compartment syndrome. The skin envelope is left open to be grafted later. Reperfusion of an extremity after prolonged ischemia can lead to accumulation of toxic metabolites and muscle necrosis. Systemic acidosis and shock can occur when venous flow is reestablished after several hours of muscle ischemia. Appropriate multidisciplinary management of reperfusion injury should be undertaken with the anesthesia and critical care teams. Rhabdomyolysis and myoglobinuria may result from such injuries and may potentially lead to cardiac arrhythmias or renal failure.

With improvements in microsurgical techniques, replantation of major amputations of the upper extremity has a high rate of technical success. The long-term results, however, are largely unchanged since the original descriptions. In cases where a large crush component exists, the functional outcome is generally poor. Even in the best of circumstances, multiple revisional surgeries and extensive therapy are necessary. A poor functional outcome must be weighed against an amputation and prosthesis. Our patient was able to demonstrate weak pinch grip and flexion of the wrist and digits at 12 months with minimal extension. Hot and cold sensation was present in the fingertips with sensation to light touch in the median nerve distribution. A subsequent wrist arthrodesis was performed for a flexion contracture.

Although composite tissue allotransplantation is a possibility in this current era, replantation of a patient's amputated part is still the best option. However, injury to the amputated part from the initial trauma may impair long-term function. Improved immunosuppression protocols may potentially favor allotransplantation in the future.

## Figures and Tables

**Figure:**